# Caring for Patients With Opioid Use Disorder: What Clinicians Should Know About Comorbid Medical Conditions

**DOI:** 10.1176/appi.prcp.20180005

**Published:** 2018-12-05

**Authors:** Deepika E. Slawek, Tiffany Y. Lu, Benjamin Hayes, Aaron D. Fox

**Affiliations:** ^1^ Department of Medicine Albert Einstein College of Medicine Bronx NY

**Keywords:** Substance‐Related and Addictive Disorders, Opioids, HIV/AIDS, Hepatitis C, Hypogonadism, Opioid Overdose

## Abstract

Opioid use disorder (OUD) is a growing problem, with opioid‐involved overdose deaths quadrupling since 1999 in the United States. This article reviews comorbid medical conditions related to OUD, starting with complications of behaviors associated with opioid use (e.g., injection drug use), followed by conditions stemming from the direct effects of opioids (e.g., hypogonadism). HIV and hepatitis C virus (HCV) are common infections in people with OUD, and treatment for these conditions can be safely provided regardless of ongoing substance use. Complications of drug injection, such as HIV, HCV, skin and soft tissue infections, and infective endocarditis, may be prevented through provision of sterile syringes and supervised injection facilities. Rare, life‐threatening bacterial infections may present with signs and symptoms that mimic intoxication, such as malaise or stupor, and should be assessed in patients with fever or positive blood cultures. In addition, chronic opioid exposure can lead to hypogonadism, opioid‐induced hyperalgesia, sleep‐disordered breathing, and potentially increased risk of cardiovascular disease and neurocognitive impairment. Pharmacotherapies for OUD (buprenorphine, methadone, and naltrexone) are safe and effective and their adverse opioid effects can be managed in clinical practice. Awareness of OUD‐associated medical conditions and their treatments is an important step in improving the health and wellness of people with OUD.

**Publisher's Note:** This article is part of a special issue titled “The Opioid Crisis: Filling in the Picture.”

Opioid‐involved overdose deaths in the United States have more than quadrupled since 1999 in three interrelated waves: first, deaths involving prescription opioids increased; next, heroin‐related deaths increased; and then after 2014, mortality related to illicit synthetic opioids (e.g., fentanyl) skyrocketed (1). Preventing opioid overdose is a priority; however, as opioid use and opioid use disorder (OUD) have increased, understanding and managing medical conditions associated with OUD have become critical too.

The *DSM‐5* gives 11 criteria for OUD, two or more of which must be met for a diagnosis of OUD. These criteria emphasize continuous and compulsive opioid use leading to negative social, occupational, psychological, and physical consequences, including comorbid medical conditions (2). Among the at least 2.1 million individuals with OUD in the United States, much variability exists in demographic characteristics, method of opioid use, and attendant risks (3); data representing the entire population are limited. During the current opioid crisis, HIV and hepatitis C virus (HCV) outbreaks have garnered national attention, while other comorbid conditions, such as tobacco‐related illnesses, remain under recognized and undertreated in this population (4, 5, 6, 7). In this review, conducted by searching PubMed and relevant clinical guidelines, we present the epidemiology, clinical course, and recommended screening, treatments, and preventive interventions for the most severe and frequently occurring comorbid medical conditions of OUD.

Common OUD‐associated medical conditions may result from behaviors (e.g., injection drug use) related to opioid use or from the direct pharmacologic effects of opioids (e.g., hypogonadism). This distinction is important, because it highlights that much of the harm of illicit opioid use comes from unsafe injection practices, which could be addressed through harm reduction interventions, such as distributing sterile syringes or encouraging less risky routes of opioid administration. Additionally, some direct opioid effects can cause harm whether the use is illicit or prescribed, and these harms can be detected and managed with careful clinical monitoring. Thus, this review provides information that will be useful for both clinical care and policy development.

OUD‐associated behaviors that can lead to medical complications include mode of opioid administration (injecting, sniffing, smoking), comorbid substance use disorders, and sexual risk behaviors. Behaviors associated with OUD also may have social consequences, such as incarceration or homelessness, which affect comorbid medical conditions and access to care. In‐depth discussion of these social consequences, however, is beyond the scope of this review.

## Complications of Injection Behaviors

Injection drug use (IDU) causes common and serious OUD complications. Injection can be a private or communal behavior, and the process of preparing and injecting drugs presents several distinct risks. Commonly injected opioids include heroin, which comes as a white powder, darker brown powder, or a sticky black tar variety; illicit synthetic opioids, such as fentanyl or similar analogs; and crushed or dissolved prescription opioids, such as oxycodone or oxymorphone. Powdered opioids and other illicit substances may be cut with readily available adulterants (8). Adulterants that dilute the effects of opioids, such as caffeine, quinine, and sugar, may have health consequences when injected (8). Adulterants that increase opioid effects, such as fentanyl, have been implicated in the recent overdose increases (9). Syringes, cookers, water, and filters used in preparing opioids for injection also may be involved in infectious disease transmission, particularly when shared or reused or if nonsterile (10).

People who inject drugs may prefer injecting into a vein (mainlining), muscle (muscling), or subcutaneous or intradermal tissue (skin‐popping) (11). Each site of injection carries different risks. The relative risk of overdose through opioid injection has been found to be 15.9 times that of other routes of administration (12), and intramuscular or subcutaneous injection has been found to increase risk of soft tissue infection (11, 12). Parenteral injection may introduce bacteria beneath the skin and into the bloodstream from contaminated injection supplies, nonsterile water, or unsterilized skin (13).

Medical complications of IDU include HCV, HIV, and bacterial infection, such as skin and soft tissue infection (SSTI) or infective endocarditis (Table 1). Despite increased awareness regarding HIV transmission, access to sterile syringes and injection supplies in some areas, and other prevention efforts, rates of HCV and HIV infection remain higher among people who inject drugs than in the general U.S. population (14). In addition, SSTIs and infective endocarditis are serious complications that are increasingly frequent causes of preventable hospitalizations (13, 14).

**Table 1:** Select medical conditions comorbid to opioid use disorder, by system and route of administration

Type of condition	Conditions associated with injection drug use	Conditions associated with smoking/inhalation	Conditions associated with intranasal insufflation
**Cardiovascular**	Endocarditis	—	—
**Cancer**	Hepatocellular carcinoma (caused by Hepatitis C)	—	—
**Infectious disease**	Hepatitis A, B, and C; HIV; soft tissue infections (abscess, cellulitis, necrotizing fasciitis); infective endocarditis; septic arthritis; osteomyelitis; brain abscess; pulmonary infections; tuberculosis; tetanus	Hepatitis A, B, C; pulmonary infections; tuberculosis	Hepatitis A, B, C; pulmonary infections; tuberculosis
**Neurologic**	—	Progressive spongiform leukoencephalopathy	—
**Respiratory**	Pneumonia, septic pulmonary embolism, talc granulomatosis, emphysema, pulmonary hypertension, pulmonary edema, pneumothorax	Asthma exacerbation, emphysema, pulmonary edema, pneumothorax	Asthma exacerbation, sinus problems, hypersensitivity pneumonitis, nasal septum necrosis or perforation
**Renal**	Focal glomerular sclerosis, glomerulonephritis, nephrotic syndrome, chronic kidney disease	—	—

Adapted with permission from Ries RK, Fiellin DA, Miller SC, Saitz R (eds): The ASAM Principles of Addiction Medicine, 5th ed. Philadelphia: Wolters Kluwer, 2014, Table 72‐1

### HCV

#### Epidemiology.

In the United States, approximately 5 million people are living with HCV, making it the most common bloodborne infection (15). IDU accounts for at least 60% of acute HCV infections (5, 14, 16). Since 2004, acute HCV cases have increased nationally, with the largest increases occurring east of the Mississippi and in central Appalachia among individuals younger than age 30 (5). HCV is highly infectious through blood exposure. Within five years of IDU initiation, more than one in five people become infected with HCV (17).

#### Clinical course.

Acute HCV infection is usually asymptomatic, but patients may develop flu‐like symptoms, myalgias, or dark urine (17). Of those acutely infected, two‐thirds develop chronic HCV infection, which increases risk of long‐term sequelae, including hepatic fibrosis, cirrhosis, and hepatocellular carcinoma (18). Symptoms of chronic infection can include fatigue, arthralgias, and sicca syndrome. Chronic HCV also is associated with chronic kidney disease, diabetes mellitus, porphyria cutanea tarda, hypothyroidism, and cryoglobulinemic vasculitis (17). Patients with chronic HCV have a lifespan that is on average two decades shorter than those without chronic hepatitis (19). Highly effective medications are available, yet deaths attributable to HCV continue to rise, making HCV the most fatal infectious disease in the United States (20).

#### Screening.

HCV complications may not develop for decades, underscoring the importance of HCV awareness, prevention, and screening. Guidelines from the American Association for the Study of Liver Diseases and the Infectious Diseases Society of America recommend that all people who inject drugs be offered HCV testing annually (16). People using intranasal drugs also should be offered periodic HCV testing, because sharing contaminated implements for nasal inhalation can transmit HCV (16). Point‐of‐care testing with oral swab and finger stick are available and could be implemented in substance use disorder treatment and mental health settings (21, 22).

#### Treatment.

New direct‐acting antiviral therapies for HCV have high cure rates (>90% in many populations) and are better tolerated than older treatments, such as interferon. Referral for treatment should happen at the time of diagnosis (18). Typically, evaluation involves determining HCV genotype, viral load, hepatic function, amount of fibrosis, and the patient's psychosocial readiness for treatment (16). A fibrosis score can now be determined with noninvasive testing; however, liver biopsy may be necessary in some cases. Guideline‐recommended treatments vary based on HCV genotype, presence of cirrhosis, and prior treatment history; common regimens are one pill daily for as few as 8–12 weeks (16) (see Table 2 for key resources and guidelines).

**Table 2:** Key resources and guidelines for care of patients with opioid use disorder and comorbid medical conditions

Resource	Organization	URL
Guideline for Prescribing Opioids for Chronic Pain	Centers for Disease Control and Prevention	https://www.cdc.gov/drugoverdose/prescribing/guideline.html
HCV Guidance: Recommendations for Testing, Managing, and Treating Hepatitis C	American Association for the Study of Liver Diseases and Infectious Diseases Society of America	www.hcvguidelines.org
HIV/AIDS	U.S. Department of Health and Human Services	aidsinfo.nih.gov
Pre‐Exposure Prophylaxis (PrEP) Guidelines	Centers for Disease Control and Prevention	www.cdc.gov/hiv/risk/prep
Sexually Transmitted Diseases Treatment Guidelines	Centers for Disease Control and Prevention	www.cdc.gov/std/treatment/default.htm
Syringe Exchange	North American Syringe Exchange Network	http://nasen.org

Clinical trial data demonstrate that patients infected with HCV and receiving OUD treatment can achieve high HCV cure rates regardless of ongoing drug use (23). Therefore, patients with OUD should not be excluded from HCV treatment or clinical trials (15). Although ongoing IDU increases risk for reinfection, treating people who are at high risk of transmitting HCV infection to others—treatment as prevention—could prevent new HCV infections (24).

### HIV

#### Epidemiology.

By the end of 2013, 1.1 million people were living with HIV/AIDS, and IDU was the mode of transmission for 103,000 men and 68,000 women in the United States (25). From 2010 to 2014, IDU accounted for 11% of new HIV infections among men and 23% of new HIV infections among women (25). Although HIV incidence among people who inject drugs is below the peak rates of the early 1980s, increasing IDU caused by the current opioid crisis puts at least 220 U.S. counties at high risk for HIV outbreaks (26). People younger than age 30 who inject drugs are at highest risk for HIV infection because they are more likely than their older counterparts to share injection equipment and to participate in high‐risk sexual behavior (27).

#### Clinical course.

HIV infection can be categorized as asymptomatic, symptomatic, or AIDS, based on symptoms and CD4+ T‐lymphocyte concentration (CD4 count) (28). Signs and symptoms of acute infection commonly include fever, fatigue, night sweats, truncal maculopapular rash, headache, lymphadenopathy, pharyngitis, myalgia, arthralgia, weight loss, diarrhea, depression, and oral or genital ulcers (29). These symptoms may last from fewer than 14 days to 10 weeks. Other, less common presentations of acute HIV infection include leukopenia, thrombocytopenia, and elevated liver function. Signs and symptoms of acute HIV infection are commonly missed because of their nonspecific nature. People who inject drugs and others who participate in HIV risk behaviors (e.g., having multiple sexual partners, exchanging sex for money or drugs, engaging in receptive anal intercourse) who present with these symptoms may require HIV testing.

Standard HIV diagnostic testing relies on detection of anti‐HIV antibodies, which may not be detectable for up to 15–20 days following infection, even when using the most recently developed assay (30). During this initial period, diagnosis can be made by directly testing for the presence of viral ribonucleic acid (30). After the acute infection, an asymptomatic phase may last for years. As the CD4 count gradually decreases, the immune system weakens, increasing risk for atypical infections, such as oral thrush or shingles, and generalized symptoms, such as persistent diarrhea or fever. AIDS is diagnosed when the CD4 count falls below 200 cells/μL or when an AIDS‐defining illness, such as *Pneumocystis jiroveci* pneumonia, esophageal candidiasis, or Kaposi sarcoma, is present.

#### Screening.

Guidelines from the Centers for Disease Control (CDC) recommend at least annual HIV screening for people who inject drugs and other people with OUD and elevated HIV risk (31, 32). It is recommended that initial HIV testing be performed with a U.S. Food and Drug Administration (FDA)–approved fourth–generation antigen/antibody immunoassay, which detects HIV‐1 and HIV‐2 antibodies and HIV‐1 p24 antigen. A positive fourth–generation assay requires additional antibody immunoassay testing to differentiate between HIV‐1 and HIV‐2. When results from these two tests conflict, further testing with an HIV‐1 nucleic acid test is recommended (33). However, some sites of care serve people who may not be able to follow up and, therefore, need immediate results. Point‐of‐care testing or self‐testing with oral fluids or dried blood spots may be implemented in substance use disorder treatment programs or sites providing emergency care (34). Fourth‐generation point‐of‐care assays have up to 95% sensitivity and 100% specificity and can provide results within 20 minutes (35). People who are newly diagnosed as having HIV should be linked to medical care and counseling as soon as possible (31).

#### Treatment.

During the past 25 years, improvements have been made in HIV diagnostic tests, antiretroviral therapy, and patient longevity. Life expectancy for those who start treatment early is nearly equal to that of the general population. Despite medical advances, people who inject drugs, nonwhites, and other marginalized groups have experienced smaller reductions in HIV‐related mortality (36). Some clinicians withhold treatment from people who inject drugs because of concerns about incomplete medication adherence; however, decreased pill burden or supportive interventions, such as pillboxes, reminders, or directly observed therapy, can improve adherence (37). Antiretroviral treatment initiation is recommended as soon as possible after diagnosis regardless of CD4 count. This treatment reduces the likelihood of transmission to others and improves long‐term health outcomes (32).

HIV treatment guidelines published by the U.S. Department of Health and Human Services can be found online at aidsinfo.nih.gov (38) (Table 2). Some first‐ and second‐line regimens contain strong CYP3A4 inhibitors but may interact with medications commonly prescribed for other medical or psychiatric diagnoses (38). Some nonnucleoside reverse transcriptase inhibitors (e.g., efavirenz) interact with methadone and often require an increase in methadone to avoid opioid withdrawal symptoms (38). This interaction can be avoided with the newer once‐daily tablets (38). Medication lists should be reviewed for potential interactions (38). First‐line treatment regimens for HIV infection are listed (Table 3). Adherence to antiretroviral regimens is critical for suppressing viral load and has significantly improved with one‐pill‐once‐daily regimens and decreased pill burden (39).

**Table 3:** Recommended initial antiretroviral regimens for treatment of HIV^a^

Antiretroviral regimen	No. of pills
Abacavir/Lamivudine/Dolutegravir	1
Tenofovir/Emtricitabine + Dolutegravir	2
Elvitegravir/cobicistat/Tenofovir/Emtricitabine^b^	1
Tenofovir/Emtricitabine + Raltegravir	2
Bictegravir/Emtricitabine/Tenofovir	1

a Adapted from aidsinfo.nih.gov (38).

b Contains a CYP3A4 inhibitor.

#### Prevention.

Many evidence‐based HIV prevention interventions exist, including condom distribution, partner notification and agreements, and harm reduction practices (see below). Pharmacologic HIV prevention is also possible with preexposure prophylaxis (PrEP) using a combination tenofovir disoproxil fumarate/emtricitabine pill taken once daily. PrEP has been shown to prevent HIV infection among people who inject drugs (40). The CDC recommends offering PrEP to adults ages 18 years or older without established HIV infection who have injected any nonprescribed drug in the prior six months and meet one of the following additional criteria: any sharing of injection or drug preparation equipment during the past six months, enrollment in a methadone or buprenorphine treatment program in the past six months, history of anal sex without condom use, history of sexually transmitted infection (STI) during the past six months, ongoing relationship with an HIV‐positive partner, or infrequent condom use during sex (41) (Table 2).

### SSTIs

#### Epidemiology.

SSTIs are the most common reason for hospital admission among people who inject drugs (42). Hospitalization for SSTIs among people who inject drugs doubled between 1993 and 2010 (42). Female gender, intramuscular or subcutaneous injection, more frequent injection, using mixtures of heroin and cocaine (speed balls), and using black tar heroin are associated with increased infection risk (43). Injecting crushed prescription opioids also may lead to adverse skin reactions and limb ischemia due to talc or other inactive ingredients in tablets (44).

#### Clinical course and treatment.

The spectrum of SSTIs and their sequelae includes abscesses, cellulitis, systemic sepsis, necrotizing fasciitis, pyomyositis (abscesses of skeletal muscle), abscesses of visceral organs, bone/joint infections, and endovascular infections. Abscesses are the most frequent SSTI among people who inject drugs and present as red, tender fluctuant areas in the skin (45). The most important aspects of abscess care are cleaning the infected area, incision, and drainage (13). Patients with SSTIs may present with nonspecific symptoms, such as fatigue, sweating, or impaired mental status, which could be mistakenly attributed to opioid intoxication or withdrawal rather than to acute infection (13).

Necrotizing fasciitis, a serious SSTI involving deep layers of subcutaneous tissue, has a high incidence among people who inject drugs. Severe pain out of proportion to the skin appearance, rapid spread of skin erythema, or unstable vital signs can be a clue to a more serious infection. Necrotizing fasciitis is a medical emergency and often requires advanced imaging, surgical exploration, debridement of infected skin and muscle, and intravenous antibiotics (13).

Most SSTIs are caused by commensal flora, but contaminated drugs, drug adulterants, or drug use paraphernalia also may introduce bacteria (13). *Staphylococcus aureus* or *Streptococcus* species most frequently cause SSTIs; however, outbreaks of less common organisms, such as *Pseudomonas* or *Clostridium* species may occur among people who inject drugs. Prevalence of methicillin‐resistant *Staphylococcus aureus* is elevated among this population and is of concern because of its virulence and association with more severe infections, such as necrotizing fasciitis (46). Documented outbreaks from lethal *Clostridium* species associated with black tar heroin have been associated with wound botulism, tetanus, and necrotizing fasciitis (13). Botulism is uncommon, however, and signs and symptoms, such as difficulty swallowing, slurred speech, blurred vision, and muscle paralysis, may be confused with intoxication.

### Endovascular Infections

#### Epidemiology.

Endovascular infections, including infective endocarditis, septic thrombophlebitis, and mycotic aneurysms, are risks of IDU. Lifetime prevalence of infective endocarditis among people who inject drugs ranges from 0.5% to 12% and is among the most serious infectious complications of IDU (45). Infections at other sites, skin colonization with *Staphylococcus aureus*, and a history of infective endocarditis increase risk of infective endocarditis in this population (13). HIV infection also predisposes people who inject drugs to infective endocarditis, with a fourfold increase in incidence in those with versus without HIV infection shown in one study (47).

#### Clinical course and treatment.

Signs and symptoms of endovascular infection include fever, night sweats, joint and muscle pain, and weight loss, or may be more severe, with heart failure, cardiac arrhythmias, abscesses of visceral organs, and immune‐mediated phenomena. Fever and a new heart murmur should prompt additional investigation via blood cultures and echocardiogram. Endovascular infection should be ruled out in those with bacteremia who inject drugs (13). Treatment consists of antibiotics and sometimes surgery.

### Other Bacterial Infections

Pulmonary infections, including pneumonia, tuberculosis (TB), and septic emboli also are common complications of IDU (48). TB is highly transmissible through respiratory droplets, making outbreaks most common in communal settings lacking adequate ventilation. Exposure to TB may occur in jails or homeless shelters, and HIV infection is a risk factor for TB. Pneumonia may occur via hematogenous spread to the lungs. Aspiration pneumonia could be caused by opioid‐induced stupor and suppressed cough reflex. Impaired immune defenses from other comorbid conditions also can lead to atypical pneumonias. In addition to illicit IDU, prescribed opioid use has been associated with increased risk of pneumonia (49, 50).

Injecting crushed prescription opioids may introduce other risks. Talc from crushed oxycodone, buprenorphine tablets, or other adulterants may lead to foreign‐body granulomatosis of the lung, liver, or other organs. Injected particles lodge in pulmonary capillaries, leading to an immune reaction, and may subsequently cause fibrosis, emphysema, and pulmonary hypertension. Patients present with cough, shortness of breath, and increased sputum production, but chest x‐ray may appear normal, making diagnosis difficult (48).

### Harm Reduction Practices for IDU

Many comorbid conditions associated with IDU could be prevented through harm reduction practices. Distribution of sterile syringes and other injection supplies through syringe exchange programs reduce reuse and sharing of syringes and thus decrease HIV and HCV transmission and SSTI risk. Researchers have demonstrated that sterile syringe services reduce HIV and HCV transmission without increasing frequency of injection (51, 52, 53). Resources describing local syringe exchange programs are available through the North American Syringe Exchange Network (Table 2). Where available, supervised injection facilities or safe drug consumption sites provide a space where people can bring drugs to inject more safely (54). Typically, these spaces offer well‐lit stalls, sterile injection equipment, and medical supervision supplied to reduce risk of HIV transmission, SSTIs, and overdose (54). Provision of intravenous heroin or another potent opioid in a medical setting has been shown to reduce use of illicit drugs and may reduce mortality (55). Heroin replacement therapy is available in Europe and Canada (56).

## Complications of Intranasal Opioid Insufflation

Another common mode of opioid administration is intranasal insufflation (sniffing) of powdered or dissolved opioids. Less of the drug is bioavailable via intranasal administration; therefore, overdose risk is lower than that for injection (57). Intranasal opioid use is a risk factor for HCV infection, but the precise mechanism of HCV transmission is unknown (16). Intranasal insufflation can lead to other comorbid conditions (Table 1), including asthma exacerbation, sinus problems, hypersensitivity pneumonitis, or nasal septum necrosis or perforation (58).

## Complications of Inhalation of Combusted Smoke

Smoking heroin or other opioids is less common in the United States than in Asia or Western Europe. Powdered heroin may be smoked in cigarettes or heated on foil, so the fumes can be inhaled through a tube (“chasing the dragon”). Although overdose risk appears to be lower with heroin inhalation in comparison to intravenous use (57), respiratory illnesses present serious health risks. Respiratory disease associated with inhalation include asthma exacerbation, pneumothorax, pulmonary edema, and early‐onset emphysema (59) (Table 1).

Inhaling opioids also may cause extrapulmonary complications. Inhaling heated heroin vapor has been associated with progressive spongiform leukoencephalopathy, which may injure neurons in the cerebral cortex and cerebellum. Patients may present with slurred speech, ataxia, tremors, or neurobehavioral symptoms. Although incidence is rare, 25% of reported cases have been fatal. It is unclear whether the toxic insult is from heroin itself or an unknown contaminant (60).

## Comorbid Conditions Associated With Co‐occurring Behaviors

### Concomitant Substance Use Disorders

Many people with OUD have other substance use disorders that can lead to significant medical complications. Among U.S. residents (2011–2013) reporting past‐year heroin use and other past‐year substance use disorders, more than 33% used alcohol, 25% used cocaine, 25% used cannabis, and nearly 50% used prescription opioids (61). Polysubstance use is associated with substantial morbidity and mortality. Benzodiazepine use disorder is common among people with OUD and dramatically increases overdose risk (62). Alcohol use disorder is associated with accidents, peptic ulcer disease, liver disease, cancers, and hypertension (48). Alcohol use disorder co‐occurring with OUD increases the risk of overdose and should be screened for in patients with known OUD (63). Cocaine use disorder is linked to cardiovascular disease, particularly stroke and myocardial infarction, as well as chronic kidney disease (64). Cannabis use disorder, although often considered more benign, may be associated with accidents, respiratory conditions, and adverse cognitive effects (65). Awareness of medical conditions associated with other substance use disorders is important in the delivery of care for OUD patients.

Tobacco‐related illnesses also disproportionately affect people with OUD. Cohort studies suggest that half of people with a substance use disorder die from tobacco‐related illnesses, and the death rate of smokers is four times greater than that of nonsmokers over long periods of follow‐up (66). Prevalence of tobacco use may be as high as 93% among patients in methadone maintenance treatment programs (67). Chronic medical conditions stemming from tobacco use, such as cardiovascular disease, stroke, and cancer, are leading causes of death among people with OUD (68). Patients receiving OUD treatment seldom receive evidence‐based smoking cessation interventions despite the high burden of tobacco‐related illness. Nicotine replacement therapy and varenicline are effective treatments for tobacco use disorder among patients maintained on methadone (69, 70). Implementing systematic interventions at OUD treatment programs to increase access to smoking cessation treatments should be a high priority.

### Sexually Transmitted Infections (STIs)

People who use drugs have higher STI rates than the general population (14). Intoxication may influence decisions regarding sexual activity, including condom use, which may lead to gonorrhea, syphilis, herpes, chlamydia, HIV, or other STI exposure. Additionally, people who exchange sex for money or drugs are at risk for STIs, and they may be exposed to threatening situations where they are unable to control sexual risk behaviors (71).

## Comorbid Conditions Due to the Direct Effects of Opioids

By any route of administration, opioids may result in adverse health effects. Opioids cause respiratory depression and can lead to unintended overdose. Opioid receptors are ubiquitous throughout the body; therefore, chronic opioid exposure may have other effects, such as hypogonadism, worsening pain, sleep‐disordered breathing, cardiovascular disease, and neurotoxicity. The direct effects occur whether opioids are prescribed or taken illicitly, but these effects usually do not limit OUD treatment. The opioid agonist treatments buprenorphine and methadone are safe and effective, and fear of side effects should not prevent eligible patients from starting possibly life‐saving treatment. Overdose risk is lower with buprenorphine than with other opioids. Buprenorphine has only partial agonist activity at the mu‐opioid receptor, and high doses typically do not lead to life‐threating respiratory depression (e.g., the ceiling effect). The opioid antagonist treatment naltrexone does not exert direct opioid effects, but advantages and disadvantages of this treatment option compared to opioid agonist treatments are beyond the scope of this review.

### Unintended Overdose

The risk of unintended opioid overdose increases with co‐occurring medical and psychiatric conditions. Opioids have a dose‐dependent effect on respiratory depression. Patients with OUD often have chronic pain, anxiety, and other mood disorders, for which they may be prescribed sedating medications (antidepressants, antipsychotics, mood stabilizers, sleep aids) that further exacerbate respiratory depression (72, 73). Most notably, concurrent use of benzodiazepines and opioids is a major risk factor for overdose (74, 75). The cumulative effects of multiple sedating medications with opioid use increase overdose risk (73). Chronic liver and/or pulmonary disease, which may impair opioid metabolism and pulmonary reserves, may worsen opioids’ respiratory depression effects (76).

Evidence‐based strategies can reduce unintended overdose among patients taking illicit or prescribed opioids. Maintenance treatment with buprenorphine or methadone for patients with OUD is associated with large reductions in overdose mortality (77, 78). The CDC guidelines on prescribing opioids for chronic pain recommend strategies for reducing overdose risk for patients prescribed chronic opioid therapy (76). Clinicians can provide overdose prevention education and prescribe naloxone, an opioid antagonist used to temporarily reverse overdose (76).

### Chronic Pain

A complex, bidirectional relationship exists between opioid use and chronic pain. Approximately 40% of patients in methadone maintenance programs have chronic pain (79). People with OUD frequently have chronic pain for several reasons: chronic pain may have preceded OUD or stem from conditions associated with OUD, such as HIV or HCV; accidents and trauma may occur because of OUD; and opioid‐induced hyperalgesia may occur secondary to chronic opioid exposure. The risk for developing OUD among patients who start prescription opioids for chronic pain is likely small but significant (80). Among patients with OUD taking prescription opioids, pain is a leading motivation for use (81). Opioid use is also associated with increased risk of accidents and trauma (82), which can lead to chronic pain. Lastly, opioids can induce hyperalgesia, in which pain sensitization is enhanced despite opioid therapy. The prevalence of opioid‐induced hyperalgesia is unknown, and no standardized criteria for diagnosis exist (83, 84). However, in contrast to pain from injuries, opioid withdrawal, or physiologic tolerance, pain symptoms from opioid‐induced hyperalgesia paradoxically improve when the opioid dosage is reduced.

Chronic pain can be difficult to treat in people with OUD. Comprehensive multimodal approaches should be used, including OUD treatment; nonopioid analgesics; nonpharmacologic treatments, such as physical therapy and acupuncture; and other psychosocial treatments directed at pain and comorbid mental health conditions (85). Multidisciplinary treatment teams with pain, addiction, and mental health specialists are likely best equipped to care for patients with OUD and chronic pain (86).

### Hypogonadism

Clinically significant endocrine dysfunction due to hypogonadism occurs in an estimated 9%‐29% of patients (87) who use chronic opioids (88). Opioids directly suppress the hypothalamic‐pituitary‐gonadal and ‐adrenal axes, subsequently reducing production of estrogen, testosterone, and other sex hormones (89). Patients may experience decreased libido, erectile dysfunction, irregular or absent menses, infertility, anxiety, depression, and fatigue (90). Chronic opioid use is associated with low bone density, likely due to hypogonadism, leading to increased risk of osteoporosis and fractures (90, 91). No consensus exists for the management of opioid‐induced hypogonadism, but options include hormone supplementation with testosterone or estrogen, opioid rotation, and opioid reduction or discontinuation (92). No clear recommendations have been developed for screening, prevention, or management of opioid‐associated osteoporosis (93).

### Sleep‐Disordered Breathing

Sleep‐disordered breathing occurs in as many as 70%–85% of people who use opioids (94). Central sleep apnea is the most common type of sleep‐disordered breathing associated with opioid use, likely because opioids depress hypoxic and hypercapnic ventilatory drives. Obstructive sleep apnea also may be worsened by opioid‐induced suppression in airway muscle activity (95). Sleep‐disordered breathing adversely affects daytime alertness and cognition and may increase long‐term risk of cardiovascular disease and mortality. Reducing the opioid dosage may improve sleep‐disordered breathing, although such a reduction may not be indicated for patients receiving opioid agonists for OUD. Noninvasive positive‐pressure ventilation is an effective treatment for sleep‐disordered breathing (96).

### Other Potential Toxicities

Chronic opioid use is associated in some cohort studies with increased risk of cardiovascular disease and mortality, including myocardial infarction and stroke (96, 97). The mechanism of increased risk is likely multifactorial, including co‐occurring risk factors, such as tobacco use, and possibly direct opioid effects. In addition, methadone can alter electrical conduction within the cardiac tissue, as evidenced by QTc prolongation on electrocardiogram that can lead to fatal arrhythmias. Recommendations for cardiac screening prior to methadone treatment initiation include taking a history of personal or familial heart disease, assessing cardiac risk factors and use of QTc‐prolonging drugs, and performing an electrocardiogram for some patients (98, 99). Opioids also may affect cognitive function. Chronic opioid use has been associated with deficits in working memory, planning, impulse control, and decision making (100).

## OUD Treatment and Comorbid Medical Conditions

Initiation of OUD treatment is an opportunity to screen for and identify comorbid medical conditions that may have gone unrecognized and untreated. The FDA has approved three medications for OUD (methadone, buprenorphine, and naltrexone), which may be offered concomitantly with care for other medical conditions. In the U.S., methadone maintenance treatment is only available in regulated opioid treatment programs. Although some studies have demonstrated improved HIV treatment outcomes with concomitant methadone maintenance for OUD, some patients continue to have difficulty with adherence to antiretroviral therapy and need more intensive interventions (e.g., directly observed therapy in which methadone and antiretroviral medications are administered simultaneously) (101, 102, 103). Buprenorphine maintenance treatment has been successfully integrated into primary care and HIV treatment settings (104) and buprenorphine treatment is associated with HIV viral load suppression, improved HCV outcomes, and identification and management of other chronic medical conditions (105, 106, 107). Fewer studies have evaluated naltrexone and management of co‐occurring medical conditions (108). Use of naltrexone has been evaluated in a pilot study of people living with HIV/AIDS who use opioids and alcohol, but the small sample size limited evaluation of HIV outcomes (11).

## Conclusions

Clinicians who care for people with OUD should be aware of acute and chronic infectious diseases (e.g., HIV, HCV, SSTI, and infective endocarditis) and chronic noninfectious conditions (e.g., sleep‐disordered breathing and hypogonadism) that are associated with opioid use. Episodes of acute medical care, such as hospitalization for overdose or infective endocarditis, provide opportunities to diagnose and initiate treatment for OUD and associated medical conditions. Likewise, initiating OUD treatment offers the opportunity to better manage comorbid conditions or provide preventive care. In both settings, people who use drugs need patient‐centered care that maintains their dignity and reduces shame. Substance use disorders are highly stigmatized in the United States, and stigma may prevent people with OUD from seeking medical care. Therefore, communications that use person‐first language (e.g., people who use drugs) and avoid stigmatizing slang (e.g., addict or drug seeker) are essential components of care. Although many common OUD‐associated comorbid conditions may be prevented through harm reduction practices, integration of medical and OUD treatment may improve health outcomes by furthering access to appropriate medical care for people with OUD. Awareness of OUD‐associated medical conditions and their treatments is an important step in improving the health and wellness of people with OUD.

**Key Points**

- HIV and HCV infection are common in patients with opioid use disorder (OUD).
- Successful treatments for HIV and hepatitis C virus (HCV) can be provided to patients with OUD, regardless of ongoing substance use.
- Complications of injection drug use, such as HIV, HCV, skin and soft tissue infections, and infective endocarditis, could be prevented with harm reduction practices (e.g., sterile syringe services and supervised injection facilities).
- Rare, life‐threatening bacterial infections may present with signs and symptoms that mimic intoxication, such as malaise or stupor, and should be thoroughly assessed in patients with fever or positive blood cultures.
- Chronic opioid exposure can lead to hypogonadism, opioid‐induced hyperalgesia, sleep‐disordered breathing, and increased risk of cardiovascular disease and neurocognitive impairment.
- Medications for OUD—buprenorphine, methadone, and naltrexone—are safe and effective, and awareness of adverse opioid effects can improve clinical practice.
